# One-Year Safety Analysis of the COMPARE-AMI Trial: Comparison of Intracoronary Injection of CD133^+^ Bone Marrow Stem Cells to Placebo in Patients after Acute Myocardial Infarction and Left Ventricular Dysfunction

**DOI:** 10.1155/2011/385124

**Published:** 2011-02-27

**Authors:** Samer Mansour, Denis-Claude Roy, Vincent Bouchard, Louis Mathieu Stevens, Francois Gobeil, Alain Rivard, Guy Leclerc, François Reeves, Nicolas Noiseux

**Affiliations:** ^1^Division de Cardiologie, Département de Médecine, Centre Hospitalier de l'Université de Montréal (CHUM), 3840, Rue Saint Urbain, Montréal, Québec, Canada H2W 1T8; ^2^Axe Cardio-Métabolique, Centre de Recherche du CHUM (CRCHUM), Montréal, Québec, Canada H2W 1T7; ^3^Département d'Hématologie, Hôpital Maisonneuve-Rosemont (HMR), Montréal, Québec, Canada H1T 2M4; ^4^Faculté de Médicine, Université de Montréal, Montréal, Québec, Canada H3C 3J7; ^5^Division de Chirurgie Cardiaque, Département de Chirurgie, Centre Hospitalier de l'Université de Montréal (CHUM), Montréal, Québec, Canada H2W 1T8

## Abstract

Bone marrow stem cell therapy has emerged as a promising approach to improve healing of the infarcted myocardium. Despite initial excitement, recent clinical trials using non-homogenous stem cells preparations showed variable and mixed results. Selected CD133^+^ hematopoietic stem cells are candidate cells with high potential. Herein, we report the one-year safety analysis on the initial 20 patients enrolled in the COMPARE-AMI trial, the first double-blind randomized controlled trial comparing the safety, efficacy, and functional effect of intracoronary injection of selected CD133^+^ cells to placebo following acute myocardial infarction with persistent left ventricular dysfunction. At one year, there is no protocol-related complication to report such as death, myocardial infarction, stroke, or sustained ventricular arrhythmia. In addition, the left ventricular ejection fraction significantly improved at four months as compared to baseline and remained significantly higher at one year. These data indicate that in the setting of the COMPARE-AMI trial, the intracoronary injection of selected CD133^+^ stem cells is secure and feasible in patients with left ventricle dysfunction following acute myocardial infarction.

## 1. Introduction

Despite improvements in survival rate after myocardial infarction (MI) with recent medical and surgical advances [1], the reduced heart function attributed to irreversible loss of viable cardiomyocytes remains a major clinical problem [2]. Intracoronary (IC) injection of progenitor cells emerged as a valuable therapeutical approach to improve healing of the ischemic heart. Despite the use of various experimental protocols with mixed cells showing mixed results [3], four recent meta-analyses of bone marrow stem cells (BMSCs) transplantation in the setting of acute MI proved the feasibility and safety and demonstrated a slight but positive functional effect [4–7]. However, there is currently uncertainty as to which of the BMSC population is most potent in stimulating angiogenesis and cardiac repair. The hematopoietic CD133^+^ cells possess high engraftment, multipotent, and angiogenic capacity and appear to be valuable for cardiac repair in experimental myocardial infarction [8–11].

In a phase I study, Bartunek et al. tested the IC injection of CD133^+^ cells in patients following acute MI and demonstrated a significant improvement of the left ventricular (LV) function and perfusion as compared to controls [12]. However, they noted in the treated group an increase of coronary events with greater in-stent proliferation and larger luminal loss in the nonstented segments of the infarct-related (IR) artery [13]. Of note, these data were obtained from retrospective analysis and they lack randomized controls. Hence, we designed the COMPARE-AMI trial, the first randomized, double-blind, placebo-controlled phase II single center study comparing the IC injection of CD133^+^ cells to placebo on the cardiac repair in patients with acute MI and persistent LV dysfunction [14, 15]. Herein, we report the one-year safety analysis on the first randomized patients.

## 2. Methods

### 2.1. Study Design and Objectives

**Figure 1:**
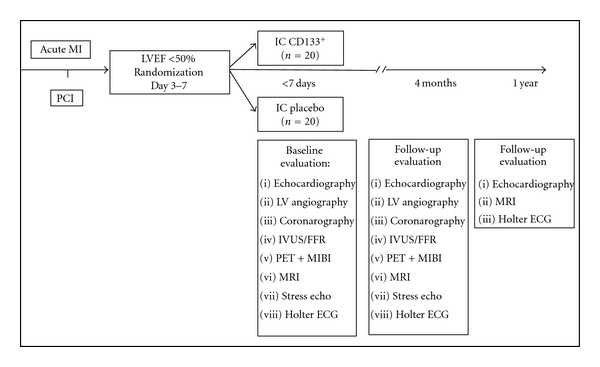
Flow chart of the COMAPRE-AMI trial. MI indicates myocardial infarction, PCI percutaneous coronary intervention, LVEF left ventricular ejection fraction, IC intracoronary, LV left ventricle, IVUS, intravascular ultrasound, FFR fractional flow reserve, PET positron emission tomography, MIBI technetium 99 m sestamibi, MRI magnetic resonance imaging, and ECG electrocardiogram.

COMPARE-AMI study is a single-center, randomized, prospective, double-arm, double-blind, placebo-controlled phase II clinical trial investigating the feasibility, safety, and efficacy of the IC administration of selected autologous CD133^+^-enriched hematopoietic BMSC versus placebo in patients following acute MI with persistent dysfunctional myocardium despite successful catheter reperfusion. The flow chart of the trial is detailed in Figure 1. The primary end point of the study is a composite of safety and efficacy end points aiming to determine the change in the coronary atherosclerotic burden progression proximal and distal to the stented segment of the culprit artery at 4 months as compared to baseline, and the change in the left ventricle ejection fraction (LVEF) at 4 months relative to baseline. The secondary end points include the occurrence of a major adverse cardiac event (MACE) defined as death, MI, stroke, and target vessel revascularisation (TVR) or the occurrence of major arrhythmias defined as sustained ventricular arrhythmia or survived sudden death. Patients are followed for 2 years, and the data will be unblinded 12 months after the randomization of the last patient.

### 2.2. Study Population and Protocol

In brief, patients between 30 and 75 years are eligible if they presented an acute ST elevation MI successfully reperfused by means of coronary stent implantation and demonstrated a substantial persistent LV dysfunction defined by a LVEF ≤50% but ≥25% on echocardiography obtained within 48 hours after the successful reperfusion therapy. Written informed consent is obtained within 7 days after the onset of acute MI if the patient is free of any exclusion criteria such as known previous MI, cardiogenic shock, chronic cardiomyopathy, liver disease, renal failure, concomitant disease with a life expectancy of less than 1 year, alcohol or drug dependency, contraindication for bone marrow (BM) aspiration, blood transfusion in the previous 24 hours, hematopoietic disease, chronic inflammatory disease, malignancy, stroke in the previous 3 months, or transient ischemic attack in the previous 24 hours. The study protocol was approved by Health Canada (CTA 113925 and ITA 123840) and by institutional ethics review board.

Until September 2009, 20 patients were successfully enrolled in the trial. A total of 18 patients completed the 4-month followup and 13 patients reached the 1-year followup. All patients underwent BM aspiration 3 to 7 days after receiving reperfusion therapy for acute MI. An average volume of 100 mL of the BM was obtained and transferred immediately to the cell processing laboratory where patients were randomly assigned to receive placebo medium (normal saline containing 10% autologous plasma) or autologous CD133^+^ cells. CD133^+^ progenitor cells for this trial are immunomagnetically selected using the CliniMACS® system (Miltenyi Biotec GmbH) according to manufacturer's protocols. Within 12 hours of BM harvest, the study suspension is sent back to the cardiac catheterisation laboratory for IC administration in the IR artery using intermittent balloon inflation as previously described in [16]. In summary, the infusate is delivered via an over-the-wire percutaneous transluminal coronary angioplasty catheter over 3 min of balloon inflation within the previously placed intracoronary stent. Three minutes of reperfusion are incorporated after each cycle of cell infusion. A total of 4 cycles are given. Patients are routinely discharged 48 hours following the infusion procedure with the usual postacute MI care medications.

Myocardial functional evaluation is done with echocardiography, cardiac magnetic resonance imaging (MRI), left ventricular angiography, echocardiography, technetium 99 m sestamibi single photon emission computed tomography, and positron emission tomography with [17] fluorodeoxyglucose at baseline and 4 months of followup. Cardiac MRI and echocardiography are repeated at 1 year of followup.

### 2.3. Safety Evaluation

To determine the safety of the IC injection of CD133^+^ cell suspension, standard laboratory tests including white blood cells (WBC) count, C-reactive protein (CRP), creatine kinase (CK), and creatine kinase-muscle and brain (CK-MB) type and troponin T measurements were performed before, 30 and 60 minutes, 6 hours, and 24 hours after the IC injections. In addition, all patients were continuously monitored until discharge and underwent 24-hour ECG monitoring at baseline, 1, 4, and 12 months after the procedure. Furthermore, a quantitative coronary angiography, pressure-derived fractional flow reserve (FFR), and an IC ultrasonography were performed in the IR artery and a remote artery at baseline and 4-month followup.

### 2.4. Statistical Analysis

Baseline demographic and clinical variables were collected for all patients. Invasive and noninvasive analyses were performed by operators blinded to all clinical and other functional data. Data are presented as mean ± standard deviation or median and interquartile range when appropriate. Continuous variables were analyzed using Student's *t*-test. Fully parameterized longitudinal mixed effect models were built for echocardiographic measurement, including a coefficient for each point in time at baseline, 4 and 12 months (the MIXED procedure in SAS software, version 9.1; SAS Institute, Cary, NC). These models account for the correlation between repeated measurements in the same patient. For all analyses, *P* < .05 were considered statistically significant.

## 3. Results

### 3.1. Clinical Characteristics

Herein, we are presenting the one-year safety analysis of the first twenty patients randomized and treated in the COMPARE-AMI trial. The demographic and clinical characteristics are showed in Table 1. The mean age was 52.2 ± 8.9 years with a predominance of males (90%) and the culprit lesion was located on the left anterior descending artery in 90%. A complete occlusion was noted in the proximal segment of the IR artery in 65% of cases. Maximum troponin T and CKMB were 10.48 ± 8.34 Ug/L and 341 ± 260.7 U/L, respectively, suggesting large infarcts. All patients underwent a successfully reperfusion with stenting using a drug-eluted stent (DES) in 75% or a bare metal stent (BMS) in 25% of cases. Adjuvant medical therapy included aspirin, clopidogrel, *β*-blockers, angiotensin-converting enzyme inhibitors, and statins.

### 3.2. Safety Analysis

BM harvests were well tolerated by all patients without any related complication. For an average of 10 × 10^6^ CD133^+^ cells, we obtained a mean recovery of more than 95% when comparing before and after cell separation. Moreover, we obtained a mean purity higher than 65% for selected CD133^+^ cells. Cell purity is validated using flow cytometric analysis using standard techniques. Cell viability is assessed by trypan bleu exclusion, which is always greater than 95%. The IC injection was performed 6.4 ± 2.2 days after stenting. Two patients had a small hematoma at the arterial access site (one radial and one femoral) that did not require any specific treatment. As compared to baseline, no significant differences in the WBC count, CRP, CK, CK-MB, and troponin T levels were found at 24 hours (Table 2). In addition, in-hospital continuous ECG monitoring did not reveal any episode of sustained ventricular arrhythmia, and only two patients had at least one episode of nonsustained ventricular tachycardia (NSVT). The serial 24-hour holter ECG monitoring performed at 1, 4, and 12 months revealed a small increase in the number of patients who had at least one run of a NSVT (five patients at 4 and 12 months as compared to two patients at baseline). In addition, despite a low number of isolated ventricular premature beats (VPB) at baseline of 6.5 [1.8, 14.8] and after treatment of 4.5 [3.0, 10.5], we observed an important increase at 1-month followup with 30.0 [13.0, 129.5]. Number of VPBs decreased thereafter at 4 months with a median of 9.0 [3.0, 117.0]. Until one-year followup, no spontaneous sustained ventricular arrhythmias or survived sudden death were noted and no patient required an implantable cardioverter-defibrillator (ICD). 

Furthermore, up to 12-month followup no MACE, angina, or stent thrombosis were reported (Table 3). During the initial hospitalization and before randomization, 2 patients had an episode of heart failure that required diuretics. Of note, one patient stopped for one week his medication and was hospitalized 3 months after his randomization for heart failure with a temporary drop of his LVEF down to 15%. Finally, at 4-month followup, restenting was necessary in 3 patients to treat in-BMS restenosis. No in-DES restenosis or de novo lesions in the IR artery were observed. Moreover, despite the significant lower pressure-derived FFR in the culprit artery as compared to the nonculprit artery at baseline:, 0.88 ± 0.05 versus 0.96 ± 0.04, *P* < .001, no significant modification was noted in the delta FFR at 4-month followup as compared to baseline in the culprit versus nonculprit artery (−3.7% ± 5.4 versus −1.1% ± 4.6 resp., *P* = .148) suggesting the absence of local or diffuse acceleration of coronary atherosclerosis.

### 3.3. LV Function Assessment after Randomization

**Figure 2:**
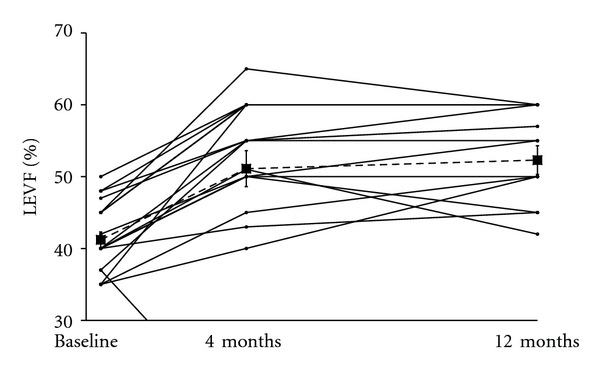
Left ventricular ejection fraction improvement assessed by echocardiography for each randomized patient at baseline before treatment (*n* = 20), 4 months (*n* = 18) and 12 months of followup (*n* = 13). Cardiac function was significantly improved at 4 and 12 months (*P* < .001). LVEF indicates left ventricular ejection fraction.

As shown in Figure 2, echocardiographic assessment of the LVEF demonstrated a significant improvement at 4-month followup (*n* = 18) as compared to baseline (51.1% ± 2.5 versus 41.2% ± 1.1 resp., *P* < .001). This improvement was sustained at 12-month followup (52.3% ± 2.0, *P* < .001 versus baseline, *n* = 13).

## 4. Discussion

In the setting of acute MI, several studies [4–7] have shown a functional benefit of IC infusion of BMSC compared with the standard treatment alone. Probably, the simplest approach to myocardial cell therapy in the clinical setting is the transfer of BM mononuclear cells into the myocardium. However, using unmodified marrow or unselected mononuclear cells can prevent the delivery of the “ideal” cell for myocardial regeneration that could be available in a small number or lost during the preparation process. In addition, few of the unselected mononuclear cells could formally meet the stem cell criteria and the vast majority are leukocytes that may primarily induce local inflammation, rendering the actual stem cell effects insignificant. Selected CD133^+^ BM cell population contains a small proportion of clonogenic cells, which have a very high potential to induce neoangiogenesis [18]. Furthermore, there is accumulating evidence that the CD133^+^/CD34^−^ subpopulation includes multipotent stem cells with a significant potential for differentiation into mesenchymal and other nonhematopoietic lineages. 

Therefore, Bartunek et al. conducted a phase I trial [12] testing the IC injection of purified CD133^+^ BMSC into the acutely infracted myocardium after successful stenting of the culprit artery. They showed a significant improvement in the LV function in parallel with an increase in the myocardial perfusion and viability suggesting a potent angiogenic effect of the injected cells. However, the following safety concerns in the cell-treated group were reported: first a small but significant rise in the CRP, second ventricular arrhythmias were observed in 4 treated patients, and third a higher rate of in-BMS restenosis and de novo lesion was found. Moreover, they raised the possibility of a “Janus-like” effect where they reported in a retrospective analysis a significant reduction of the luminal diameters and epicardial conductance of the IR artery, as assessed from the pressure-derived FFR [13] mainly in early compared to late IC CD133^+^ cells administration [17] which seems to be consistent with a higher risk for atherosclerosis progression. Yet, these data were obtained from retrospective analysis; they lack proper randomized controls and systematic use of IC ultrasound imaging to track changes in the vascular wall. 

Accordingly, we initiated the COMPARE-AMI trial, a randomized, double-blind, controlled study testing the safety, feasibility, and functional effect of the IC injection of CD133^+^ cells as compared to placebo in relatively high-risk patients with acute MI and persistent LV dysfunction. The safety and functional effect will be addressed using time frames that are consistent with clinical applicability and emerging safety profile. To date twenty patients were successfully randomized and treated, eighteen completed 4 months of followup, and thirteen of them reached one year of followup. Herein, we are presenting our first safety analysis where we did not observe any major adverse events such as death, stroke, MI, angina, heart failure, or stent thrombosis within the first 12 months following randomization. One patient stopped his medication for one week and was hospitalized for heart failure. In parallel with other trial results, [12, 16] we did not report any complication related to the BM harvest or the technique of IC infusions. Cardiac enzymes levels remained unchanged during the initial 24 hours following injection. Furthermore, and in contrary to what was reported in the Bartunek trial [12], neither the WBC counts nor the CRP levels increased. Additionally, no sustained ventricular arrhythmias were recorded and none of the randomized patients required an implantable cardioverter-defibrillator. However, the serial 24-hour ECG monitoring revealed a temporary significant increase in the number of isolated VPB at 4 months that spontaneously decreased at 12 months of followup suggesting a transient electrical irritability that could be related to the ischemic burden in the first months after the MI. Of note, only 5 patients had at least one run of NSVT until one year of followup. On the other hand, all randomized patient had a coronary angiogram control at 4 months after the IC injection; no in-DES restenosis was reported; however three over five patients treated with a BMS had a significant but asymptomatic restenosis requiring restenting with a DES because of a documented significant ischemia in the target territory. Interestingly, no de novo lesion was reported at 4-month followup, and no significant difference was found in the delta FFR as compared to baseline in the culprit versus nonculprit artery suggesting the absence of any acceleration of atherosclerosis. Finally, the 4-month echocardiography assessment showed a significant increase in the LVEF superior to what we observed in the placebo groups of the randomized trials [4–7] suggesting a potential effect on the cardiac recovery in the CD 133^+^ injected patients. 

In conclusion, the proposed experimental protocol in which IC autologous CD133^+^ cells are injected after acute MI is safe, without any serious adverse events up to one year of followup. In addition, a potential beneficial effect on the LV recovery of the enrolled patients is observed. Final results will be available 1 year after the last patient has been randomized; meanwhile the presented one-year safety results justify the continuation of the COMPARE-AMI trial. This randomized clinical study should help to support the therapeutical potential of CD133^+^ cells for the treatment of the infracted myocardium.

## Figures and Tables

**Table 1:** Demographic and clinical characteristics. BMI indicates body mass index, CAD coronary artery disease, PCI percutaneous coronary intervention, CK creatinine kinase, and CK-MB creatine kinase-muscle and brain.

Age (yrs)	52,2 ± 8.9
Male (%)	90
BMI	28 ± 5.7
Diabetes (%)	15
Hypertension (%)	35
Hyperlipidemia (%)	20
Smoking (%)	70
Family history of CAD (%)	15
Door to balloon (Min)	123.6 ± 97.4
PCI to injection (days)	6.4 ± 2.2
Killip class	I: 95; II: 5
Maximal ST elevation (mm)	11.2 ± 5.5
Q waves on admission (%)	55
Peak troponin T (UI/mL)	10.48 ± 8.34
Peak CK (UI/mL)	2744 ± 2193
Peak CK-MB (UI/mL)	341 ± 260.7

**Table 2:** Biological markers after IC injection, no change over time (All *P* = NS). WBC indicates white blood cells count, CRP C-reactive protein, CK creatinine kinase, and CK-MB creatine kinase-muscle and brain.

	Baseline (end of the IC injection)	30 min	60 min	6 hrs	Day 1
WBC	8.12 ± 1.6	8.65 ± 1.96	8.92 ± 2.31	7.74 ± 1.94	7.29 ± 1.69
CRP	11.09 ± 12.16	10.15 ± 8.56	12.15 ± 12.8	13.18 ± 14.24	13.08 ± 13.96
Troponin T	0.83 ± 1.02	0.88 ± 1.07	0.91 ± 1.13	0.8 ± 0.95	0.66 ± 0.84
CK	167.4 ± 95.6	176.5 ± 99	204 ± 135.9	171.5 ± 85.3	160.4 ± 81.97
CK-MB	46.8 ± 22.2	51.33 ± 28.17	55.56 ± 27.58	72 ± 40.28	60.7 ± 39.90

**Table 3:** In-hospital, 4- and 12-month reported adverse events. SVT indicates sustained ventricular tachycardia, ICD implantable cardioverter-defibrillator, BMS bare metal stent DES drug-eluting stent, BM bone marrow, and IC intracoronary.

	In-hospital	4 months	12 months
Mortality (*n*)	0	0	0
Re-infarct (*n*)	0	0	0
Stroke (*n*)	0	0	0
SVT/ICD (*n*)	0	0	0
Heart failure (*n*)	2	1	1
Stent thrombosis (*n*)	0	0	0
In-BMS restenosis/occlusion	0	3	0
In-DES restenosis/occlusion	0	0	0
Bleeding/transfusion (*n*)	2/0	0/0	0/0
BM harvest related (*n*)	0	NA	NA
IC Injection related (*n*)	0	NA	NA
